# Engineering of HN3 increases the tumor targeting specificity of exosomes and upgrade the anti-tumor effect of sorafenib on HuH-7 cells

**DOI:** 10.7717/peerj.9524

**Published:** 2020-07-20

**Authors:** Cong He, Doulathunnisa Jaffar Ali, Yumin Li, Yanliang Zhu, Bo Sun, Zhongdang Xiao

**Affiliations:** 1 State Key Laboratory of Bioelectronics, School of Biological Science and Medical Engineering, Southeast University, Nanjing, Jiangsu, China; 2 Key Laboratory for Developmental Genes and Human Disease, Ministry of Education, Institute of Life Sciences, Jiangsu Province High-Tech Key Laboratory for Bio-Medical Research, Southeast University, Nanjing, Jiangsu, China

**Keywords:** Exosomes, GPC3, HN3, HuH-7, HEK-293, Sorafenib

## Abstract

Safe, efficient and cancer cell targeted delivery of CRISPR/Cas9 is important to increase the effectiveness of available cancer treatments. Although cancer derived exosomes offer significant advantages, the fact that it carries cancer related/inducing signaling molecules impedes them from being used as a reliable drug delivery vehicle. In this study, we report that normal epithelial cell-derived exosomes engineered to have HN3 (HN3LC9-293exo), target tumor cells as efficiently as that of the cancer cell-derived exosomes (C9HuH-7exo). HN3LC9-293exo were quickly absorbed by the recipient cancer cell in vitro. Anchoring HN3 to the membrane of the exosomes using LAMP2, made HN3LC9-293exo to specifically enter the GPC3^+^ HuH-7 cancer cells than the GPC3^−^ LO2 cells in a co-culture model. Further, sgIQ 1.1 plasmids were loaded to exosomes and surprisingly, in combination with sorafenib, synergistic anti-proliferative and apoptotic effect of loaded HN3LC9-293exo was more than the loaded C9HuH-7exo. While cancer-derived exosomes might induce the drug resistance and tumor progression, normal HEK-293 cells-derived exosomes with modifications for precise cancer cell targeting like HN3LC9-293exo can act as better, safe and natural delivery systems to improve the efficacy of the cancer treatments.

## Introduction

The Clustered Regularly Interspaced Short Palindromic Repeats (CRISPR)-associated endonuclease (Cas) 9 is an adaptive immune system in archaea (Marraffini, 2015). It is guided by a chimeric single stranded RNA (sgRNA) and binds to the region of interest in DNA adjacent to the protospacer adjacent motif (PAM) sequence (Hsu et al., 2013). It enables insertion or deletion of DNA sequences at a particular genomic DNA locus, hence recognized as an efficient tool for the genome editing (Marraffini, 2015; Sander & Joung, 2014; Ma, Zhang & Huang, 2014). However, this genomic tool/technique is limited from being utilized for advanced clinical applications due to the lack of safe, disease specific and targeted delivery methods. Although, researchers have recently, developed several kinds of carriers, varying from viral vectors to synthetic nanoparticles (Biagioni et al., 2018; Rui, Wilson & Green, 2018; Xu et al., 2019), each has its own merits and drawbacks. For example, viral vectors have high loading capacity but are not much safe in the aspect of their clinical applicability (Huang et al., 2011; Wang et al., 2016; Nayerossadat, Maedeh & Ali, 2012). This warrant, a safe and natural delivery platform for advanced clinical applications. In light of this context, exosomes which are physiologically secreted, from cells and act as, lipid bilayer covered nano-vesicles, hold promising advantages to be used as delivery vehicles over polycationic liposomes and viral vectors (Wang et al., 2016; Nayerossadat, Maedeh & Ali, 2012; Van Niel, D’Angelo & Raposo, 2018). They bud from the internal vesicles of multivesicular bodies and are involved in cell to cell communication by transferring functional materials between cells (Van Niel, D’Angelo & Raposo, 2018; Lee, El Andaloussi & Wood, 2012; Sun et al., 2013; Raposo & Stoorvogel, 2013). This study primarily focuses on generating a natural delivery platform for CRISPR with normal cells derived exosomes and testing its targeted therapeutic efficacy on hepatocellular carcinoma (HCC).

Hepatocellular Carcinoma (HCC) leads to the second most cause of cancer-related death globally (Müller, Bird & Nault, 2020; Sia et al., 2017). Sorafenib, a small molecule has been shown to exert a potent anti-tumor growth in vitro in various types of cancer and an effective approved drug for liver cancer treatments at present (Gauthier & Ho, 2013). Still, its therapeutic effects have been reported to be affected by several signaling pathways such as reactivation of ERK and inhibition of MAPK signaling pathways (Moritz et al., 2010; Wilson et al., 2012). Several studies report potential sorafenib resistance mechanisms, and there is a need for combination therapy for HCC treatment (Zhu et al., 2017; Desai et al., 2017).

IQ-domain GTPase-activating proteins (IQGAPs) are a conserved family of proteins in eukaryotes (Noritake et al., 2005). Among the different types of protein, IQGAP1 has been linked to the progression of several cancers including liver cancer (Johnson & Sharma, 2009; Sanchez-Laorden, Viros & Marais, 2013). Inhibition of IQGAP1 has been shown to affect the proliferation of cancer cells and thus acts as an effective target in cancer therapeutics (Zoheir et al., 2016; Su, Liu & Song, 2017). It has been reported to induce the nuclear localization of *β*-catenin in wnt/ *β*-catenin pathway and activate transcription of wnt target genes (eg. cyclin D1 and c-Myc) (Goto et al., 2013). Overexpression of IQGAP1 might contribute to constitutive activation of wnt signaling and thus leads to cancer progression. Studies indicate targeting wnt signaling pathway can induce apoptosis in melanoma via caspase activation (Tarapore et al., 2010). Hence, disruption of IQGAP1 could suppress wnt signaling via inhibiting translocation of *β*-catenin and induce apoptosis by activating caspase cascade.

Glypican-3 (GPC3), belongs to heparan sulfate (HS) proteoglycans family. It is anchored to the cell surface by glycosyl-phosphatidylinositol (Filmus & Selleck, 2001). Even though it is expressed in fetal liver, it has not been reported to be identified in adult hepatic tissue. It is frequently noted to be elevated in HCC and identified as a biomarker of HCC diagnosis and prognosis (Nakatsura et al., 2014). Recently, anti-GPC3-CAR-T has been developed and used to suppress liver tumor progression (Gao et al., 2014; Jiang et al., 2016). Thus it is considered to be an active target in HCC treatments (Zhang et al., 2012; Baumhoer et al., 2008; Yamauchi et al., 2005). HN3 is a human antibody targeting GPC3 (Feng & Ho, 2014) with high affinity (Feng et al., 2013). Recently, it has been found that the growth of HepG2 and Hep3B generated tumor xenografts can be inhibited with the treatment of HN3 (Gao et al., 2015). The effective applicability of HN3 antibody to eliminate liver cancer cells in clinical trials remains under active investigation (Wu, Liu & Ding, 2016). Thus we hypothesized that fusing of human antibody HN3 with epithelial cell-derived exosomes can improve its tumor targeting efficiency as GPC3 is specifically over expressed in liver cancer cells.

Recently, cancer–derived. exosomes have been shown to have unique advantages for delivery vehicles as they can exert cell specific tropism (Kim et al., 2017). As it also acts as carriers for cancer related molecules, its therapeutic application has to be precisely controlled (Hu et al., 2018; Penfornis et al., 2016). Thus, this study aims to find a safe, efficient and tumor specific delivery platform of CRISPR-Cas9, an effective gene editing tool. Herein, to confer tumor specificity, HEK293 cells were stably expressed with HN3 protein, an antibody to specifically target tumor liver cells. To confer efficient cleavage, Cas9 protein was also stably expressed in the same cells. The exosomes secreted from this engineered HEK293 cells (HN3LC9-293) were further electroporated with sgIQ 1.1, a sgRNA to direct Cas9 protein to the site of IQGAP1 in the genomic locus for efficient cleavage. In addition, this study aims to investigate the synergistic cytotoxic effect of sorafenib, with sgIQ 1.1 loaded engineered HN3LC9-293 exosomes (HN3LC9-293exo) to achieve effective anti-tumor efficacy in liver cancer for future clinical applications.

## Materials & Methods

### Cell culture

HEK-293, HuH-7 and LO2 cell lines were purchased from the American Type Culture Collection (ATCC). The cells were cultured in Dulbecco’s modified eagle’s medium (DMEM), supplemented with 10% fetal bovine serum (FBS) and 1% of penicillin/streptomycin (100 units/mL penicillin and 100 µg/mL streptomycin) and maintained in a humidified chamber at 37 °C and 5% CO_2_. All cell culture reagents were obtained from Hyclone Laboratories Inc. (Logan, UT, USA). Fetal bovine serum was serum-depleted by passing through a 0.22 µm steritop filter (Millipore, USA) followed by an overnight ultracentrifugation at 110,000 g.

### In vitro T7E1 assay

The Cas9 and sgRNA plasmids were transiently transfected into HuH-7 cells using jetPRIME polyplus transfection reagent (Polyplus Transfection, France), as per the protocol given by the manufacturer. The cells were harvested 48 h later and the genomic DNA was isolated using Multisource Genomic DNA Miniprep Kit (Axygen, USA). The sgRNA genomic target site was PCR amplified with specific primers (Table S1). The PCR amplicons were purified, 150 ng of DNA was re-annealed using a thermocycler and then digested with T7 endonuclease I (T7E1, NEB, USA) according to the manufacturer’s instructions. The digested DNA was analyzed with Tanon-4200 Chemiluminescent Imaging System and band intensities were quantified using ImageJ software.

### Isolation of exosomes

The culture media of C9HuH-7, LC9-293 and HN3LC9-293 cells were collected and the exosomes were isolated as previously described (Zhou et al., 2017). Total protein was isolated from 20 µL of exosomes using RIPA buffer (Beyotime Biotechnology, China) and quantified using Micro BCA protein assay kit (CoWin Biotechnology, China) as per the manufacturer’s protocol.

### Characterization of engineered exosomes

Purified 100-fold diluted engineered exosomes (C9HuH-7exo, LC9-293exo and HN3C9-293exo) were used to analyze the size distribution by DLS (Zetasizer Nano ZS, Malven Instruments, UK) method. TEM (JEM-2100. JEOL, Japan) was used to observe the morphology of the engineered exosomes. Purified exosomes were transferred onto a carbon-coated grid, kept at room temperature for 20 min and then visualized under TEM.

To detect exosome-specific markers, three different exosomes pellets were lysed separately with RIPA buffer containing protease inhibitors (CoWin Biotechnology, China), isolated protein were quantified using Micro BCA protein assay kit, equal amount of protein samples were separated in SDS-PAGE, transferred to a PVDF membrane and blocked with blocking buffer. To detect the exosome protein CD63, AcGFP fusion protein and the presence of Cas9, the membrane was incubated with primary anti-CD63 (Cat#sc-5275, Santa cruz, 1:1000), anti-AcGFP (Cat#TA180011, ORIGENE, 1:1000) and anti-Flag (Cat#SAB4200071, Sigma-Aldrich, 1:1000) respectively, followed by HRP-conjugated anti-mouse IgG antibody (Cat#ab6728,Abcam, 1:500) and detected using a Tanon-4200 Chemiluminescent Imaging System.

### Labeling and cellular uptake of engineered exosomes

The fluorescent dye 1,1′-dioctadecyl-3,3,3′,3′-tetramethylindodicarbocyanine, 4-chlorobenzenesulfonate salt (DiD) was obtained from Biotium (USA) and used to label C9HuH-7exo, LC9-293exo and HN3LC9-293exo. Purified engineered exosomes were incubated with 5 mM DiD at 37 °C for 30 min in dark, and centrifuged at 10,000g for 15 min to remove the unbound dye. Labeled exosomes were then washed twice with 1X PBS and centrifuged at 100,000 g, and were resuspended in PBS prior to use.

Cellular internalization of DiD-labeled exosomes was analyzed at 3 h post-incubation using confocal microscope (Revolution XD, Andor, UK). The efficient cellular uptake of DiD-labeled exosomes was determined as well using flow cytometry (Accuri C6, Becton Dickinson Co, USA) as same as the incubation period mentioned above. In brief, after incubation with labeled exosomes, the medium was removed. To count the fluorescence labeled exosomes internalized cells, all the cells were harvested, washed twice with 1X PBS and then resuspended separately in 500 µL of 1X PBS. Cells which absorbed the labeled exosomes acquired red fluorescence and thus can be detected by flow cytometer. The detected positive cells were further analyzed with BD Accuri C6 software.

To check the specific cellular uptake of exosomes, about 3 × 10^5^ GPC3^−^ LO2 cells and 3 × 10^5^ GPC3^+^ mcherryHuH-7 cells were seeded in 12-well plates. When the cells reached about 70% confluency, HN3LC9-293exo and LC9-293exo were added directly to the cells respectively. After 4 h incubation at 37 °C, the cells were harvested, washed with PBS, analyzed by flow cytometry (BD Accuri C6, USA) and inverted fluorescence microscopy (Nikon, Japan).

### Loading of engineered exosomes with sgRNA plasmid

To load sgRNA plasmid in C9HuH-7exo and HN3LC9-293exo, DNA was transfected using 1,000 V, 10 ms, 2 pulses by Neon electroporation system as published (Kim et al., 2017). 30 µg of exosomes were mixed with 10 µg of DNA for electroporation.

### In vitro anti-tumor activity assays

HuH-7 cells were seeded at a density of 2 × 10^4^ cells/well in a 96-well microtiter plates and allowed to attach overnight. About 30 µg of C9HuH-7exo and HN3LC9-293exo encapsulated with 10 µg of sgRNA plasmid were added to the cells, with or without 8 µM sorafenib (Beyotime Technology, China) for 48 h. HuH-7 cells without any exosome formulations and engineered exosomes without sgRNA encapsulation were used as control. CCK-8 kit (Dojindo, Japan) was used to determine cell viability by measuring the absorbance at 450 nm. Data are representative of three experiments.

The effect of different exosome formulations on apoptosis was assessed using Annexin V-FITC/PI kit (Multisciences, China). In brief, HuH-7 cells were seeded in 6-well plates (2 × 10^5^ cells per well) and incubated at 37 °C for 24 h. Cells were treated with different formulations of exosomes and incubated for 48 h. After incubation, cells were collected and apoptosis was assessed according to the manufacturer’s protocol. The stained HuH-7 cells were further analyzed by Accuri C6 (BD Biosciences, CA) using CFlow (BD Biosciences, CA) software. The cells were set as positive depending on the fluorescence intensity of Annexin V-FITC or PI. Annexin V-FITC positive stain indicates, that cells are in early stage of apoptosis marked by externalization of phospholipid phosphatidylserine (PS) on cell surface. PI positive cells indicate that their cell membrane is damaged, as a consequence of cells being in end stage of apoptosis, in necrosis or dead.

### Western blot

HuH-7 cells were seeded at a density of 1 × 10^6^ cells per well in 6-well plates and incubated at 37 °C. About 24 h later, the cells were introduced with different formulations of exosomes for 48 h. Total protein was isolated and western blot was performed with primary antibodies IQGAP1 (Cat#ab133490, Abcam, 1:1000 dilution), BAX (Cat#ab32503, Abcam, 1:1000 dilution), Caspase3 (Cat#ab13847, Abcam, 1:1000 dilution) and BCL2 (Cat. #ab32124, Abcam, 1:1000 dilution), as mentioned in ***Characterization of engineered exosomes.*** HRP-conjugated anti-rabbit IgG antibody (Cat#ab6721, Abcam, 1:500 dilution) was used as secondary antibody here. GAPDH was used as an internal control (anti-GAPDH, Cat#sc-47724, Santa Cruz, 1:500 dilution).

### Statistical analysis

Data are presented as the mean ± standard deviation (SD). Student’s t-test was performed to determine the significance among different treatment groups. A value of *p* < 0.05 was considered to be significant.

## Results

### Characterization of engineered exosomes

**Figure 1:**
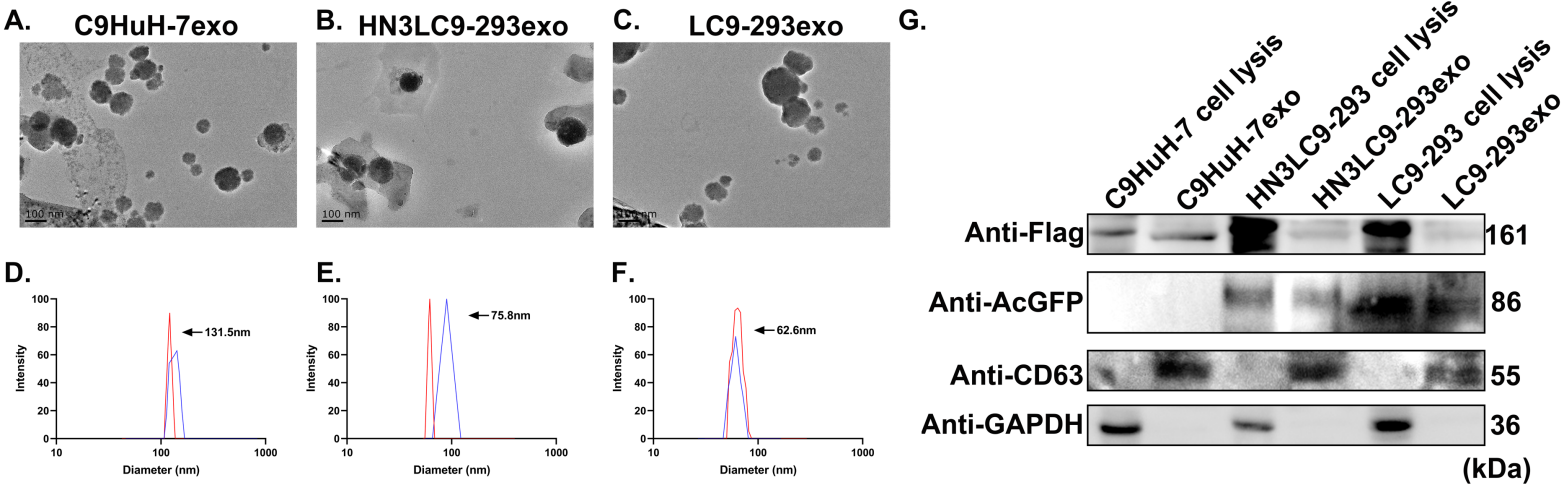
Characterization of tumor and epithelial cell-derived exosomes. (A–C) Morphology of C9HuH-7exo, LC9-293exo and HN3LC9-293exo was analyzed using TEM. (D–E) Size distribution of three engineered exosomes was analyzed by DLS. (G) Flag (tagged with Cas9 protein), AcGFP (fused with HN3-LAMP2/LAPM2) and exosomes marker protein CD63 were detected in cell and engineered exosomes lysates by western blotting. A total of 50 µg of cell and exosomes lysate was used.

To confer efficient DNA cleavage, HEK-293 and HuH-7 cells were stably expressed with Cas9. To confer targeting capabilities, HN3 human antibody sequences were fused to the extra-exosomal N terminus of human LAMP2, a protein abundantly expressed in the membranes of the exosomes, and then cloned into pLVX-AcGFP-N1, named pHN3-LAMP2-AcGFP (Fig. S1). LAMP2-AcGFP plasmid without HN3 antibody sequence was also prepared to be used as control. Cas9 expressing HEK293 cells were then stably transduced with the lentivirus vector encoding HN3-LAMP2-AcGFP and LAMP2-AcGFP plasmid to get pure lines of HN3-LAPM2-AcGFP/Cas9 and LAMP-AcGFP/Cas9 expressing HEK293 cells, named HN3LC9-293 and LC9-293 cells, respectively. Cas9 expressing HuH-7 cells were named C9HuH-7 cells and were not stably expressed with HN3-LAMP2-AcGFP, as the exosomes secreted by the cancer cells could able to possibly exert the cancer cell specific tropism (Kim et al., 2017). To start with the study, the exosomes were isolated from HN3LC9-293, LC9-293 and C9HuH-7 cells and henceforth denoted as HN3LC9-293exo, LC9-293exo and C9HuH-7exo, respectively. They were then characterized by DLS and TEM. TEM analysis showed that all the engineered exosomes were membrane surrounded, round-shaped nanovesicles with the diameters of 50 to 200 nm (Figs. 1A–1C). Size distribution was found to be approximately 100 to 200 nm for all the exosomes with DLS (Figs. 1D–1F). Furthermore, they were validated by western blot for the presence of exosomes protein CD-63, AcGFP (to confirm the expression and presence of HN3-LAMP2-AcGFP and LAMP2-AcGFP fusion protein) (Fig. 1G). They were also analyzed with anti-Flag antibodies which has been fused with the Cas9 encoding gene. As shown in Fig. 1G, exosomes marker protein CD-63 and overexpressed fusion protein LAMP2 were detected in both HN3C9-293exo and LC9-293exo lysates. Simultaneously, the presence of Cas9 protein in all the three exosomes and cell lysates were confirmed by western blot.

### HN3LC9-293exo in vitro targeting efficiency

**Figure 2:**
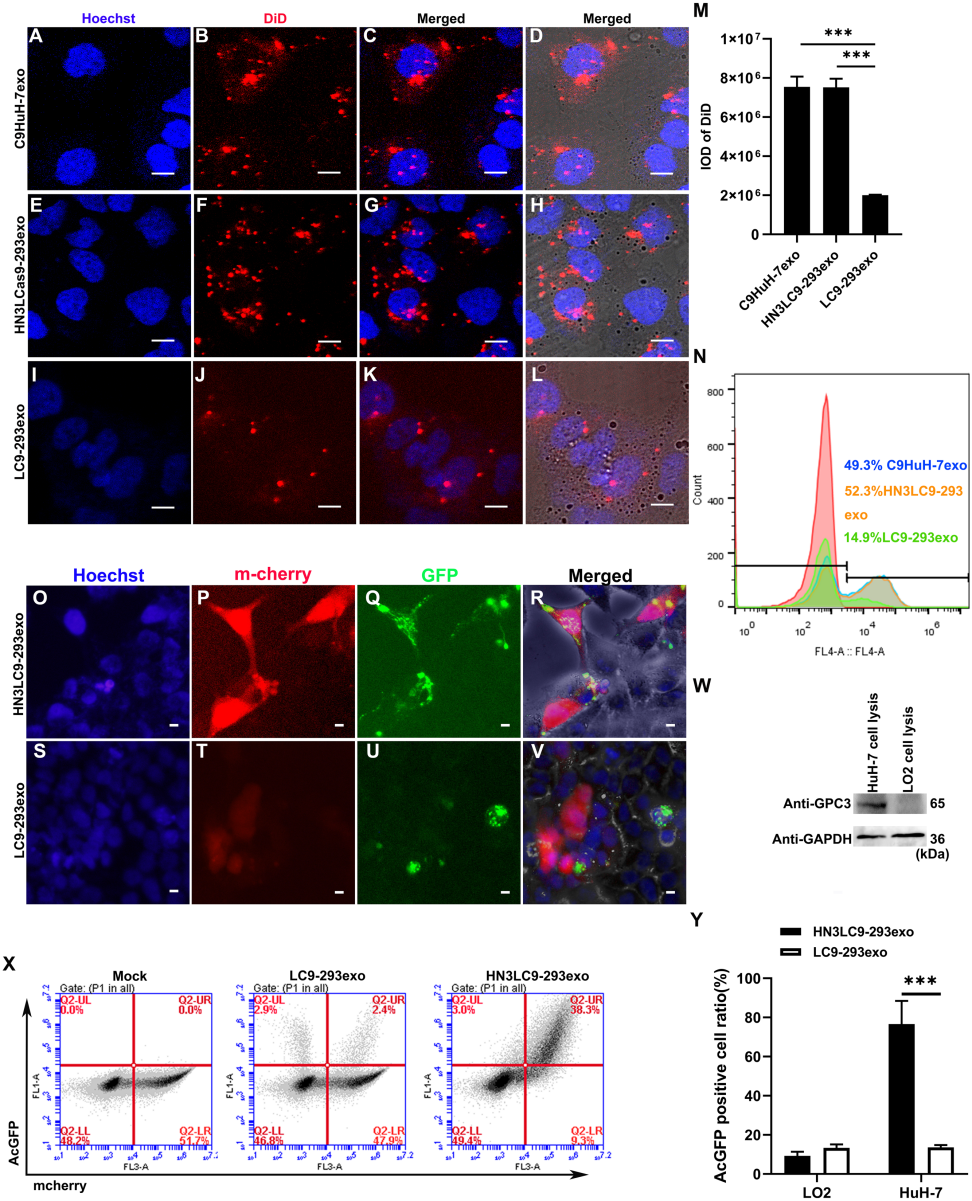
Exosomes-mediated in vitro cellular uptake and cancer cell specific targeting. (A–L) Confocal images exhibited the cellular internalization C9HuH-7exo, LC9-293exo and HN3LC9-293exo in vitro. (M) IOD (Integrated optical density) of DiD signal in respective confocal images. (N) Flow cytometry (FACS) analysis showed in vitro cellular uptake of the Did-(red), labeled C9HuH-7exo, LC9-293exo and HN3LC9-293exo at 3 h post-treatment. (O–V) Inverted fluorescence microscopy images of GPC3^+^ cancer cell specific targeting of HN3LC9-293exo and LC9-293exo at 3 h post treatment with GPC3^+^ mcherryHuH-7 cells and GPC3^−^ LO2 cells co-culture model. Red shows GPC3^+^ mcherryHuH-7 cells. Green represents HN3LC9-293exo or LC9-293exo. (W) Presence of GPC expression was detected in HuH-7 cells using western blot. (X) Flow cytometry analysis of co-cultured cells after incubation with HN3LC9-293exo or LC9-293exo (Y) Quantification of exosomes internalization based on flow cytometry analysis. Scale bars indicate 1 µm. Data are expressed as mean ± SD. *n* = 3; ***p < 0.001.

Researches revealed that nanoscale carriers transport drugs or any other agents to tumor sites due to enhanced permeability and retention (EPR) effect (Shi et al., 2017). In addition, rate of absorption for smaller exosomes by recipient cells is much quicker than larger exosomes (Caponnetto et al., 2017). As the first step to analyze whether the exosomes isolated in this study could act as a suitable delivery vehicle, cellular internalization was investigated. This is an important step in the delivery of exosome contents to the recipient cells. Cellular internalization of fluorescently-labeled C9HuH-7exo, LC9-293exo and HN3LC9-293exo at 3 h was investigated using confocal microscopy and FACS. Confocal microscopic images showed that C9HuH-7exo and HN3LC9-293exo were efficiently taken up by the recipient HuH-7 cells than the LC9-293exo (Figs. 2A–2M). To further analyze the results, the DiD-labeled fluorescence signals were quantified simultaneously by FACS (Fig. 2N). In consistent with the confocal images, the additional fluorescence signals were found and were almost similar in HuH-7 cells treated with C9HuH-7exo and HN3LC9-293exo. On the other hand, the fluorescence signals were found to be very less in the HuH-7 cells treated with LC9-293exo (normal HEK-293 cell-derived exosomes without HN3 fusion as control). Based on the cancer cell specific tropism the fluorescently labeled C9HuH-7exo might have been internalized by HuH-7 cells faster than the LC9-293exo. Interestingly, similar to C9HuH-7exo, HN3LC9-293exo was also internalized by HuH-7 cells faster than the control LC9-293exo. Even though control LC9-293exo and HN3LC9-293exo have almost same particle size (Fig. 1D–1F), the fusion of HN3 in this exosome might have caused the quicker targeting ability to the GPC3 overexpressed HuH-7 cells.

In order to analyze the potential targeting ability of HN3C9-293exo in vitro, mcherryHuH-7 cells (GPC3^+^ cells) were co-cultured with LO2 cells (GPC3^−^ cells) (Fig. 2W) and were then exposed to HN3LC9-293exo and LC9-293exo for 3 h. Confocal microscope results showed that HN3LC9-293exo were absorbed more efficiently by GPC3^+^ mcherryHuH-7 cells than GPC3^−^ LO2 cells (Figs. 2O–2V). In contrast, LC9-293exo did not show target specificity towards GPC3^+^ mcherryHuH-7 cells. Furthermore, cancer cell specific tropism of HN3LC9-293exo was quantitatively analyzed by flow cytometry (Figs. 2X & 2Y). The AcGFP containing GPC3^+^ mcherryHuH-7 cells increased up to 76.7% whereas only 11.3% AcGFP positive cells were found in the LO2 cells while treating the co-culture model with HN3LC9-293exo. In contrast, the percentage of AcGFP positive cells increased only up to 13.6% in GPC3^+^ mcherryHuH-7 cells and 13.4% in LO2 cells while treating the co-culture model with LC9-293exo. This result demonstrates that HN3LC9-293exo with GPC3 targeting HN3 antibody on its surface could be well recognized by the extracellular region of GPC3 on the cell membrane of GPC3^+^ mcherryHuH-7 cells and thus has the more cancer cell targeting specificity than LC9-293exo.

### HN3LC9-293exo function as effective natural carriers

**Figure 3:**
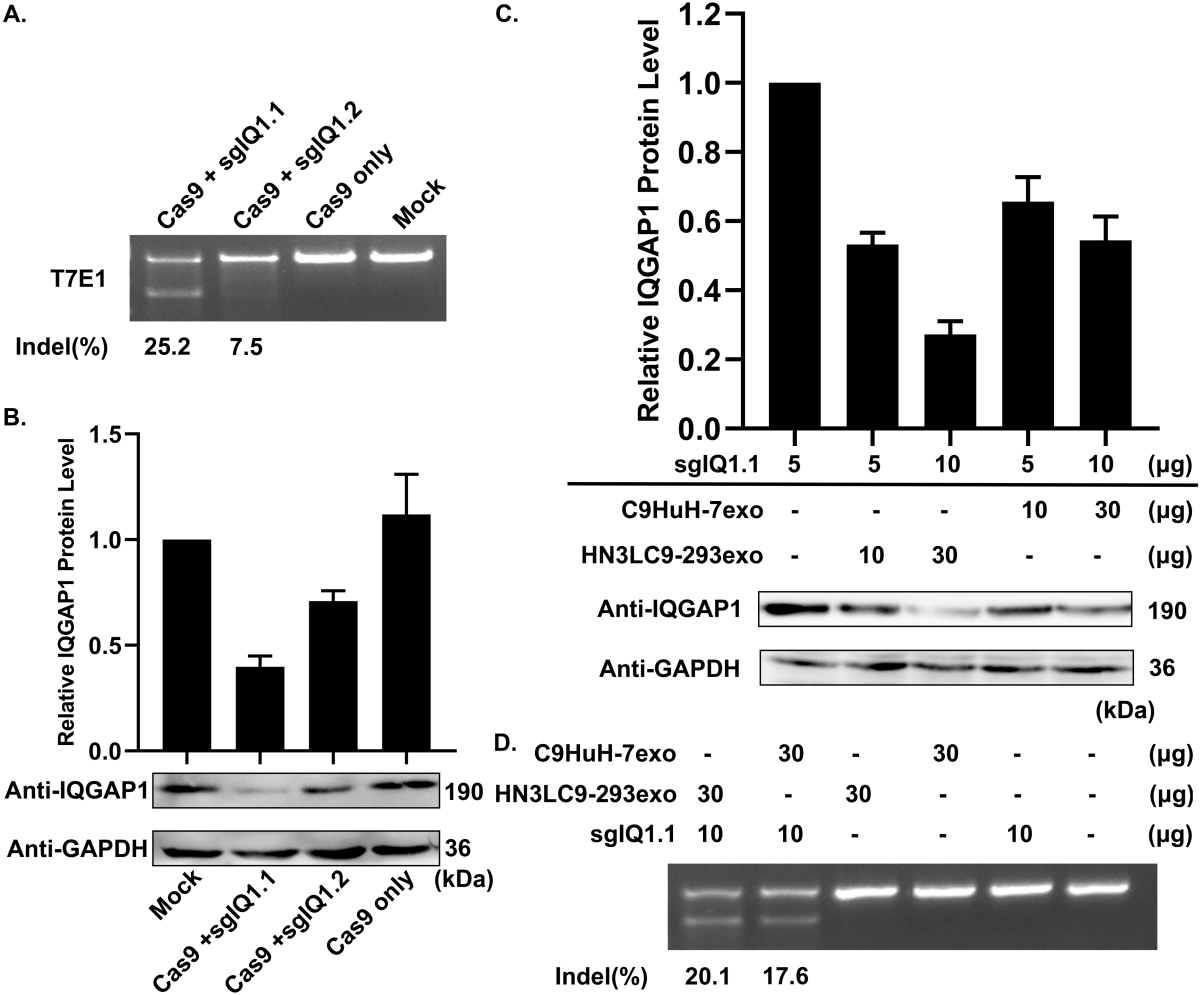
Suppression of IQGAP1 expression by the delivery of sgIQ 1.1—loaded two C9HuH-7exo or HN3LC9-293exo. (A) Cas9: sgRNA—mediated modifications in HuH-7 cells IQGAP1 expression level was analyzed. Indels were detected in HuH-7 cells using T7E1 assay to find appropriate sgRNA to be used (B) Corresponding protein reduction was assessed by western blotting. (C) Effect of indicated concentrations of engineered exosomes and DNA (via electroporation) was analyzed on IQGAP1 suppression. (D) Indel mutations were detected using a T7E1 assay (10 µg of sgIQ 1.1 and 30 µg engineered exosomes).

As the main goal of this study is to utilize the above engineered exosomes to deliver CRISPR/Cas9 to treat HCC, next we designed two different sgRNA sequences against two different sites of human locus IQGAP1 and named sgIQ 1.1 and sgIQ 1.2, respectively (Fig. S2). To analyze the cleavage efficiency at particular targeted site, an in vitro T7E1 assay was conducted on Cas9/sgRNA expression vector transfected HuH-7 cells. Cas9/sgIQ 1.1 transfection of HuH-7 cells resulted in more efficient gene editing with 25.2% as compared to Cas9/sgIQ 1.2 which showed 7.5%. On the other hand, there were no cleaved fragments in Cas9/sgRNA- treated cells (Fig. 3A). This was further confirmed with protein expression by western blot. In consistent with T7E1 assay, western blot analysis (Fig. 3B) showed that Cas9/sgIQ 1.1 transfection resulted in higher reduction in protein expression in HuH-7 cells when compared to Cas9/sgIQ 1.2 treated cells. Hence, sgIQ 1.1 alone was used further in this study.

To investigate the efficiency of HN3LC9-293exo as a potential delivery system similar to the cancer cell-derived C9HuH-7exo for HuH-7 cancer cells, both HN3LC9-293exo and C9HuH-7exo were loaded with sgIQ 1.1 via electroporation (1000 V, 10 ms, 2 pulses) (Kim et al., 2017). Varied concentrations of engineered exosomes and sgRNA plasmids were tested via electroporation to evaluate the inhibition level of IQGAP1 by sgIQ 1.1 loaded C9HuH-7exo and HN3LC9-293exo (Fig. 3C). Further, to examine the gene editing by sgIQ 1.1 loaded C9HuH-7exo and HN3LC9-293exo, in vitro T7E1 assay was performed. Interestingly, 20.1% and 17.6% indels were detected (Fig. 3D) when 10 µg of sgIQ 1.1 was electroporated into 30 µg of HN3LC9-293exo and C9HuH-7exo, respectively. These results proved that similar to the tumor-derived C9HuH-7exo, HN3LC9-293exo also could deliver the plasmid DNA into the recipient cancer cells via their HN3-GPC3 mediated target specificity and thus act as effective natural carriers for CRISPR/Cas9 delivery platform.

### sgIQ 1.1 loaded HN3LC9-293exo with sorafenib has more synergistic tumor cells killing effect

**Figure 4:**
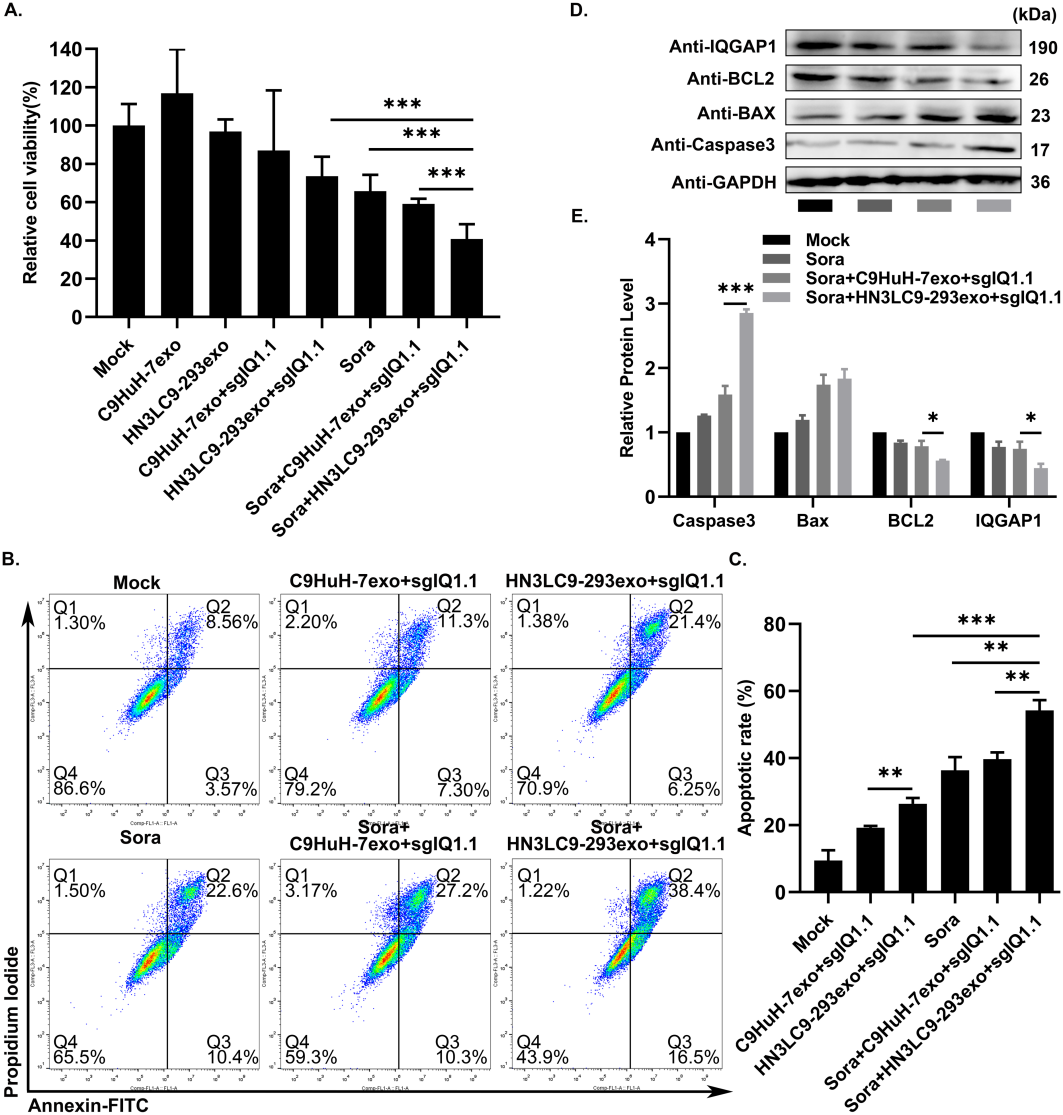
A combination therapy of engineered exosomes/CRISPR/Cas9-mediated IQGAP1 inhibition and sorafenib treatment. (A) Cell viability of cancer cells was measured using a CCK-8 assay. (B&C) Apoptosis was analyzed using an Annexin V/PI staining kit. A sorafenib concentration of 8 µM was used. Sora represents sorafenib. (D&E) Altered expression level of apoptosis markers Caspase3, BCL2 and BAX were analyzed by western blotting assay. GAPDH was used as an internal control.

Combination therapy is a flourishing approach as its synergistic effects could able to positively upgrade the effectiveness of the cancer treatment (Kim et al., 2017; Talia Golan et al., 2015). Sorafenib has been generally shown to have an excellent anti-tumor effect in various types of cancers including HCC (Scott et al., 2004; Chang et al., 2007), whereas IQGAP1 mediated ERK activation may interfere with its therapeutic action (Hedman, Smith & Sacks, 2015). Thus, in this study, the synergistic effects of sgIQ 1.1 loaded C9HuH-7exo and HN3LC9-293exo with sorafenib was investigated in HuH-7 HCC cells. HuH-7 cells were treated with sgIQ 1.1 loaded C9HuH-7exo or HN3LC9-293exo plus sorafenib (8 µM) and examined for their synergistic anti-proliferative and apoptotic effect. CCK-8 assay showed that the inhibition of IQGAP1 through sgIQ 1.1 loaded HN3LC9-293exo plus sorafenib had more synergistic anti-proliferative effect (59.2%) than sgIQ 1.1 loaded C9HuH-7exo plus sorafenib (40.9%). Yet, they both had higher anti-proliferative effect than sgIQ 1.1+C9HuH-7exo, sgIQ 1.1+HN3LC9-293exo or sorafenib alone treatments separately (13.1%, 26.5% and 34.3%, respectively) (Fig. 4A). Further, synergistic apoptotic effect was simultaneously assessed using FITC-annexin V/ propidium iodide (PI) kit. As shown in Fig. 4B & 4C, the apoptotic portion of HuH-7 cells with separate treatment of sgIQ 1.1+C9HuH-7exo, sgIQ 1.1+HN3LC9-293exo and sorafenib was 18.6%, 27.7% and 33.0%, respectively. On the other hand, sgIQ 1.1 loaded C9HuH-7exo and HN3LC9-293exo plus sorafenib treatment reached increased apoptotic rate of 37.5% and 54.9%, respectively. Furthermore, this was evidenced by increased protein levels of proapoptotic markers BAX and Caspase 3 and decreased expression of BCL2 using western blotting. The results confirmed that the destruction of IQGAP1 with sgIQ 1.1 loaded HN3LC9-293exo plus sorafenib could induce efficient apoptosis in liver cancer cells HuH-7 (Fig. 4D & 4E).

## Discussion

EVs are naturally occurring carriers that are actively involved in cell to cell communications through delivering diverse molecules between cells (Van Niel, D’Angelo & Raposo, 2018; Raposo & Stoorvogel, 2013). Cancer cells derived exosomes exert cell dependent tropism and thus increases the effectiveness of the cancer treatment by effective targeted delivery (Kim et al., 2017). Yet, the fact that it carries cancer related signaling molecules impedes them from being used as a reliable drug delivery vehicle. GPC3, of heparan sulfate (HS) proteoglycans family, is noted to be prominent in HCC and considered to be an active target in HCC treatments (Zhang et al., 2012; Baumhoer et al., 2008; Yamauchi et al., 2005). HN3 is a GPC3 targeting human antibody which has been reported to inhibit the growth of HepG2 and Hep3B xenografts *in vivo* (Gao et al., 2015). Thus in this study, fusing of human antibody HN3 with epithelial cell-derived exosomes was examined to give an improved tumor targeting efficiency as GPC3 is specifically over expressed in liver cancer cells and also to warrant a safe, efficient delivery platform of CRISPR-Cas9, an effective gene editing tool.

Cancer stem cells are a small subpopulation of cells within tumors which are resistant to conventional chemotherapy. In liver cancer cells like HCC, a novel subpopulation of CSCs were reported to be resistant to sorafenib (Xin et al., 2013). In HCC, EpCAM has been considered as an important CSC marker and is an active target of Wnt/ *β*-catenin signaling (Yamashita et al., 2007; Yamashita et al., 2009; Yamashita et al., 2013). IQGAP1 which has been shown to be correlated with many cancers promotes *β*-catenin translocation to nucleus in HepG2 cells which in turn is involved in the Wnt signaling pathway (Goto et al., 2013). In addition, Sorafenib is an approved small molecule drug widely used in treatment of HCC. IQGAP1 has been reported to interfere with its action by mediating ERK activation. Hence, IQGAP1 is considered to be one of the important targets in liver cancer therapeutics.

Considering the challenges in successful electroporation of the plasmid DNA, stably expressed Cas9 cells were prepared in both HEK-293 and HuH-7 cells. Cas9 expressing HEK293 cells were then stably transduced with the lentivirus vector encoding HN3-LAMP2-AcGFP (in which HN3 sequences were fused to the N terminus of LAMP2) and LAMP2-AcGFP plasmid to get pure lines of HN3-LAPM2-AcGFP/Cas9 and LAMP-AcGFP/Cas9 expressing HEK293 cells, named HN3LC9-293 and LC9-293 cells, respectively. C9HuH-7, Cas9 expressing HuH-7 cells which could secrete exosomes to possibly exert the cancer cell specific tropism were not stably expressed with HN3-LAMP2-AcGFP. To initiate with the actual study, HN3LC9-293exo, LC9-293exo and C9HuH-7exo were characterized by DLS, TEM which showed that the isolated exosomes were round-shaped nanovesicles surrounded by membranes with diameters ranging from 50 to 200 nm. Additionally, they were identified to have marker protein CD-63, AcGFP (fused with HN3) and also to carry Cas9 proteins from their stable mother cells.

Then, the in vitro cellular internalization of fluorescently-labeled three types of exosomes by confocal microscopy and FACS revealed that C9HuH-7exo and HN3LC9-293exo were efficiently taken up by the recipient HuH-7 cells than the LC9-293exo and were only fewer fluorescence signals were found in the HuH-7 cells treated with control LC9-293exo. In addition, when mcherryHuH-7 cells (GPC3^+^ cells) co-cultured with LO2 cells (GPC3^−^ cells) were exposed to HN3LC9-293exo and LC9-293exo for 3 h, HN3LC9-293exo were more efficiently absorbed by GPC3^+^ mcherryHuH-7 cells than GPC3^−^ LO2 cells during which LC9-293exo did not show target specificity towards GPC3^+^ mcherryHuH-7 cells. This supports that HN3LC9-293exo with GPC3 targeting HN3 antibody on its surface could be more precisely recognized by the extracellular region of GPC3 on the cell membrane of GPC3^+^ mcherryHuH-7 cells.

Interestingly, T7E1 assays using sgIQ 1.1 loaded C9HuH-7exo and HN3LC9-293exo showed 20.1% and 17.6% indels of gene editing respectively and proved that via HN3-GPC3 mediated target specificity HN3LC9-293exo also could deliver the plasmid DNA into the recipient cancer cells as same as the tumor-derived C9HuH-7exo and thus act as effective natural carriers for CRISPR/Cas9 delivery platform.

Further, as expected, sgIQ 1.1 loaded HN3LC9-293exo + sorafenib had more synergistic anti-proliferative effect (59.2%) than sgIQ 1.1 loaded C9HuH-7exo + sorafenib (40.9%). This observed decrease in anti-proliferative effect of sgIQ 1.1 loaded C9HuH-7exo irrespective of sorafenib treatment might be due to the fact that cancer/tumor-derived exosomes in general carry tumor specific functional molecules and acts as tumor microenvironment modulators.

Still, surprisingly, the sgIQ 1.1 + HN3LC9-293exo +sorafenib treatment group showed slightly higher proliferation inhibition effect on HuH-7 cells than sgIQ 1.1 + C9HuH-7exo +sorafenib when tried with combination-based cancer therapy. Also, in apoptosis analysis sgIQ 1.1 + C9HuH-7exo +sorafenib treatment showed a little less apoptosis effect on HuH-7 cells compared to sgIQ 1.1 + HN3LC9-293exo +sorafenib as in cytotoxicity test. These results raised the question whether other cancer materials that are transferred by tumor-derived exosomes could possibly inhibit the whole effect of sgIQ 1.1 + C9HuH-7exo +sorafenib in HuH-7 cells.

Furthermore, in consistent to the above-mentioned results, the apoptosis of HuH-7 cells with sgIQ 1.1 loaded C9HuH-7exo and HN3LC9-293exo plus sorafenib treatment reached 37.5% and 54.9% respectively, which were higher than the separate treatment of sgIQ 1.1+C9HuH-7exo, sgIQ 1.1+HN3LC9-293exo and sorafenib (18.6%, 27.7% and 33.0%, respectively). Simultaneously, this was evidenced by increased protein levels of proapoptotic markers BAX and Caspase 3 and decreased expression of BCL2 using western blotting. Thus the results strongly established that sgIQ 1.1 loaded HN3LC9-293exo exhibited good anti-tumor cytotoxic effect which was further enhanced by combining with sorafenib, and thus showed flourishing synergistic tumor killing effect.

## Conclusions

In summary, in this study, we have successfully found that normal HEK-293 cells-derived exosomes engineered to express HN3 antibody could be used as safe delivery vehicles for CRISPR/Cas9 platform for cancer cell specific gene therapy. Even though, cancer-derived exosomes have the cancer cell specific tropism, due to their ability to induce the drug resistance and cancer progression further, they should be precisely controlled. Thus, this study foreshadows a better, safe and natural delivery approach for CRISPR/Cas9 based gene therapy to achieve an effective anti-tumor efficacy in future cancer treatments.

##  Supplemental Information

10.7717/peerj.9524/supp-1 Supplemental Information 1 Raw Data for Figure 1

10.7717/peerj.9524/supp-2 Supplemental Information 2 Raw Data for Figure 2

10.7717/peerj.9524/supp-3 Supplemental Information 3 Raw Data for Figure 3

10.7717/peerj.9524/supp-4 Supplemental Information 4 Raw Data for Figure 4

10.7717/peerj.9524/supp-5 Supplemental Information 5 Supplementary Method

10.7717/peerj.9524/supp-6 Table S1 Primers used in this study

10.7717/peerj.9524/supp-7 Figure S1 pLVX-HN3-LAPM2-AcGFP plasmid construction A and B represent the cloning of pLVX-HN3-LAPM2-AcGFP and pLVX-LAPM2-AcGFP. (C) The detailed sequence of HN3-LAPM2-AcGFP used to generate pLVX-HN3-LAPM2-AcGFP.

10.7717/peerj.9524/supp-8 Figure S2 sgIQ1.1/1.2 plasmid construction (A) sgIQ 1.1. and sgIQ 1.2 plasmid after cloning of sgIQ 1.1/1.2 sequences into pCas-Guide-GFP plasmid. (C) Sequences of two sgRNAs which were selected to induce Cas9:sgRNA -mediated indels.

## Additional Information and Declarations

### Data Availability

The following information was supplied regarding data availability: The raw data is available as Supplemental Files.

## References

[ref-1] Baumhoer D, Tornillo L, Stadlmann S, Roncalli M, Diamantis EK, Terracciano LM (2008). Glypican 3 expression in human nonneoplastic, preneoplastic, and neoplastic tissues: a tissue microarray analysis of 4,387 tissue samples. American Journal of Clinical Pathology.

[ref-2] Biagioni A, Laurenzana A, Margheri F, Chilla A, Fibbi G, Rosso MDel (2018). Delivery systems of CRISPR/Cas9-based cancer gene therapy. Journal of Biological Engineering.

[ref-3] Caponnetto F, Maninb I, Skrap M, Palmai-Pallag T, Loreto CD, PaoloBeltrami A, Cesselli D, Ferrari E (2017). Size-dependent cellular uptake of exosomes. Nanomedicine: Nanotechnology, Biology and Medicine.

[ref-4] Chang YS, Adnane J, Trail PA, Levy J, Henderson A, Xue D, Bortolon E, Ichetovkin M, Chen C, McNabola A, Wilkie D, Carter CA, Taylor IC, Lynch M, Wilhelm S (2007). Sorafenib (BAY 43-9006) inhibits tumor growth and vascularization and induces tumor apoptosis and hypoxia in RCC xenograft models. Cancer Chemotherapy and Pharmacology.

[ref-5] Desai JR, Ochoa S, Prins PA, He AR (2017). Systemic therapy for advanced hepatocellular carcinoma: an update. Journal of Gastrointestinal Oncology.

[ref-6] Feng M, Gao W, Wang R, Chen W, Man YG, Figg WD, Wang XW, Dimitrov DS, Ho M (2013). Therapeutically targeting glypican-3 via a conformation-specific single-domain antibody in hepatocellular carcinoma. Proceedings of the National Academy of Sciences of the United States of America.

[ref-7] Feng M, Ho M (2014). Glypican-3 antibodies: a new therapeutic target for liver cancer. FEBS Letters.

[ref-8] Filmus J, Selleck SB (2001). Glypicans: proteoglycans with a surprise. Journal of Clinical Investigation.

[ref-9] Gao H, Li K, Tu H, Pan X, Jiang H, Shi B, Kong J, Wang H, Yang S, Gu J, Li Z (2014). Development of T cells redirected to glypican-3 for the treatment of hepatocellular carcinoma. Clinical Cancer Research.

[ref-10] Gao W, Tang Z, Zhang YF, Feng M, Qian M, Dimitrov DS, Ho M (2015). Immunotoxin targeting glypican-3 regresses liver cancer via dual inhibition of Wnt signalling and protein synthesis. Nature Communications.

[ref-11] Gauthier A, Ho M (2013). Role of sorafenib in the treatment of advanced hepatocellular carcinoma: an update. Hepatology Research.

[ref-12] Goto T, Sato A, Adachi S, Iemura S, Natsume T, Shibuya H (2013). IQGAP1 protein regulates nuclear localization of beta-catenin via importin-beta5 protein in Wnt signaling. Journal of Biological Chemistry.

[ref-13] Hedman AC, Smith JM, Sacks DB (2015). The biology of IQGAP proteins: beyond the cytoskeleton. EMBO Reports.

[ref-14] Hsu PD, Scott DA, Weinstein JA, Ran FA, Konermann S, Agarwala V, Li Y, Fine EJ, Wu X, Shalem O, Cradick TJ, Marraffini LA, Bao G, Zhang F (2013). DNA targeting specificity of RNA-guided Cas9 nucleases. Nature Biotechnology.

[ref-15] Hu C, Chen M, Jiang R, Guo Y, Wu M, Zhang X (2018). Exosome-related tumor microenvironment. Journal of Cancer.

[ref-16] Huang Y, Liu X, Dong L, Liu Z, He X, Liu W (2011). Development of viral vectors for gene therapy for chronic pain. Pain Research and Treatment.

[ref-17] Jiang Z, Jiang X, Chen S, Lai Y, Wei X, Li B, Lin S, Wang S, Wu Q, Liang Q, Liu Q, Peng M, Yu F, Weng J, Du X, Pei D, Liu P, Yao Y, Xue P, Li P (2016). Anti-GPC3-CAR T cells suppress the growth of tumor cells in patient-derived xenografts of hepatocellular carcinoma. Frontiers in Immunology.

[ref-18] Johnson M, Sharma BR (2009). Henderson. IQGAP1 regulation and roles in cancer. Cellular Signalling.

[ref-19] Kim SM, Yang Y, Oh SJ, Hong Y, Seo M, Jang M (2017). Cancer-derived exosomes as a delivery platform of CRISPR/Cas9 confer cancer cell tropism-dependent targeting. Journal of Controlled Release.

[ref-20] Lee Y, El Andaloussi S, Wood MJ (2012). Exosomes and microvesicles: extracellular vesicles for genetic information transfer and gene therapy. Human Molecular Genetics.

[ref-21] Ma Y, Zhang L, Huang X (2014). Genome modification by CRISPR/Cas9. The FEBS Journal.

[ref-22] Marraffini LA (2015). CRISPR-Cas immunity in prokaryotes. Nature.

[ref-23] Moritz A, Li Y, Guo A, Villén J, Wang Y, MacNeill J, Kornhauser J, Sprott K, Zhou J, Possemato A, Ren JM, Hornbeck P, Cantley LC, Gygi SP, Rush J, Comb J (2010). Akt-RSK-S6 kinase signaling networks activated by oncogenic receptor tyrosine kinases. Science Signaling.

[ref-24] Müller M, Bird TG, Nault J-C (2020). The landscape of gene mutations in cirrhosis and hepatocellular carcinoma. Journal of Hepatology.

[ref-25] Nakatsura T, Ofuji K, Saito K, Yoshikawa T (2014). Critical analysis of the potential of targeting GPC3 in hepatocellular carcinoma. Journal of Hepatocellular Carcinoma.

[ref-26] Nayerossadat N, Maedeh T, Ali PA (2012). Viral and nonviral delivery systems for gene delivery. Advanced Biomedical Research.

[ref-27] Noritake J, Watanabe T, Sato K, Wang S, Kaibuchi K (2005). IQGAP1: a key regulator of adhesion and migration. Journal of Cell Science.

[ref-28] Penfornis P, Vallabhaneni KC, Whitt J, Pochampally R (2016). Extracellular vesicles as carriers of microRNA, proteins and lipids in tumor microenvironment. International Journal of Cancer.

[ref-29] Raposo G, Stoorvogel W (2013). Extracellular vesicles: exosomes, microvesicles, and friends. Journal of Cell Biology.

[ref-30] Rui Y, Wilson DR, Green JJ (2018). Non-Viral delivery to enable genome editing. Trends in Biotechnology.

[ref-31] Sanchez-Laorden B, Viros A, Marais R (2013). Mind the IQGAP. Cancer Cell.

[ref-32] Sander JD, Joung JK (2014). CRISPR-Cas systems for editing, regulating and targeting genomes. Nature Biotechnology.

[ref-33] Scott CC, Wilhelm M, Tang L, Wilkie D, McNabola A, Rong H, Chen C, Zhang X, Vincent P, McHugh M, Cao Y, Shujath J, Gawlak S, Eveleigh D, Rowley B, Liu L, Adnane L, Lynch M, Auclair D, Taylor I, Gedrich R, Voznesensky A, Riedl B, Post LE, Bollag G, Trail PA (2004). BAY 43-9006 exhibits broad spectrum oral antitumor activity and targets the RAF MEK ERK pathway and receptor tyrosine kinases involved in tumor progression and angiogenesis. Cancer Research.

[ref-34] Shi J, Kantoff PW, Wooster R, Farokhzad OC (2017). Cancer nanomedicine: progress, challenges and opportunities. Nature Reviews Cancer.

[ref-35] Sia D, Villanueva A, Friedman SL, Llovet JM (2017). Liver cancer cell of origin, molecular class, and effects on patient prognosis. Gastroenterology.

[ref-36] Su D, Liu Y, Song T (2017). Knockdown of IQGAP1 inhibits proliferation and epithelial-mesenchymal transition by Wnt/beta-catenin pathway in thyroid cancer. OncoTargets and Therapy.

[ref-37] Sun D, Zhuang X, Zhang S, Deng ZB, Grizzle W, Miller D, Zhang HG (2013). Exosomes are endogenous nanoparticles that can deliver biological information between cells. Advanced Drug Delivery Reviews.

[ref-38] Talia Golan EZK, Hubert A, Malka Gabai R, Hen N, Segal A, Domb A, Harari G, Ben David E, Raskin S, Goldes Y, Goldin E, Eliakim R, Lahav M, Kopleman Y, Dancour A, Shemi A, Galun E (2015). RNAi therapy targeting KRAS in combination with chemotherapy for locally advanced pancreatic cancer patients. Oncotarget.

[ref-39] Tarapore RS, Siddiqui IA, Saleem M, Adhami VM, Spiegelman VS, Mukhtar H (2010). Specific targeting of Wnt/-catenin signaling in human melanoma cells by a dietary triterpene lupeol. Carcinogenesis.

[ref-40] Van Niel G, D’Angelo G, Raposo G (2018). Shedding light on the cell biology of extracellular vesicles. Nature Reviews Molecular Cell Biology.

[ref-41] Wang L, Li F, Dang L, Liang C, Wang C, He B, Liu J, Li D, Wu X, Xu X, Lu A, Zhang G (2016). *In vivo* delivery systems for therapeutic genome editing. International Journal of Molecular Sciences.

[ref-42] Wilson TR, Fridlyand J, Yan Y, Penuel E, Burton L, Chan E, Peng J, Lin E, Wang Y, Sosman J, Ribas A, Li J, Moffat J, Sutherlin DP, Koeppen H, Merchant M, Neve R, Settleman J (2012). Widespread potential for growth-factor-driven resistance to anticancer kinase inhibitors. Nature.

[ref-43] Wu Y, Liu H, Ding H (2016). GPC-3 in hepatocellular carcinoma: current perspectives. Journal of Hepatocellular Carcinoma.

[ref-44] Xin HW, Ambe CM, Hari DM, Wiegand GW, Miller TC, Chen JQ, Anderson AJ, Ray S, Mullinax JE, Koizumi T, Langan RC, Burka D, Herrmann MA, Goldsmith PK, Stojadinovic A, Rudloff U, Thorgeirsson SS, Avital I (2013). Label-retaining liver cancer cells are relatively resistant to sorafenib. Gut.

[ref-45] Xu CL, Ruan MZC, Mahajan VB, Tsang SH (2019). Viral delivery systems for CRISPR. Viruses.

[ref-46] Yamashita T, Budhu A, Forgues M, Wang XW (2007). Activation of hepatic stem cell marker EpCAM by Wnt-beta-catenin signaling in hepatocellular carcinoma. Cancer Research.

[ref-47] Yamashita T, Honda M, Nakamoto Y, Baba M, Nio K, Hara Y, Zeng SS, Hayashi T, Kondo M, Takatori H, Yamashita T, Mizukoshi E, Ikeda H, Zen Y, Takamura H, Wang XW, Kaneko S (2013). Discrete nature of EpCAM+ and CD90+ cancer stem cells in human hepatocellular carcinoma. Hepatology.

[ref-48] Yamashita T, Ji J, Budhu A, Forgues M, Yang W, Wang HY, Jia H, Ye Q, Qin LX, Wauthier E, Reid LM, Minato H, Honda M, Kaneko S, Tang ZY, Wang XW (2009). EpCAM-positive hepatocellular carcinoma cells are tumor-initiating cells with stem/progenitor cell features. Gastroenterology.

[ref-49] Yamauchi N, Watanabe A, Hishinuma M, Ohashi K, Midorikawa Y, Morishita Y, Niki T, Shibahara J, Mori M, Makuuchi M, Hippo Y, Kodama T, Iwanari H, Aburatani H, Fukayama M (2005). The glypican 3 oncofetal protein is a promising diagnostic marker for hepatocellular carcinoma. Modern Pathology.

[ref-50] Zhang L, Liu H, Sun L, Li N, Ding H, Zheng J (2012). Glypican-3 as a potential differential diagnosis marker for hepatocellular carcinoma: a tissue microarray-based study. Acta Histochemica.

[ref-51] Zhou Y, Tian T, Zhu Y, Jaffar Ali D, Hu F, Qi Y, Sun B, Xiao Z (2017). Exosomes transfer among different species cells and mediating miRNAs delivery. Journal of Cellular Biochemistry.

[ref-52] Zhu YJ, Zheng B, Wang HY, Chen L (2017). New knowledge of the mechanisms of sorafenib resistance in liver cancer. Acta Pharmacologica Sinica.

[ref-53] Zoheir KM, Abd-Rabou AA, Harisa GI, Kumar A, Ahmad SF, Ansari MA, Abd-Allah AR (2016). IQGAP1 gene silencing induces apoptosis and decreases the invasive capacity of human hepatocellular carcinoma cells. Tumour Biology.

