# Supplementary figures and images for: Engineering of HN3 increases the tumor targeting specificity of exosomes and upgrade the anti-tumor effect of sorafenib on HuH-7 cells
Source: PeerJ. 2020 Jul 20;8:e9524. doi: 10.7717/peerj.9524 (PMC7527773; doi:10.7717/peerj.9524)

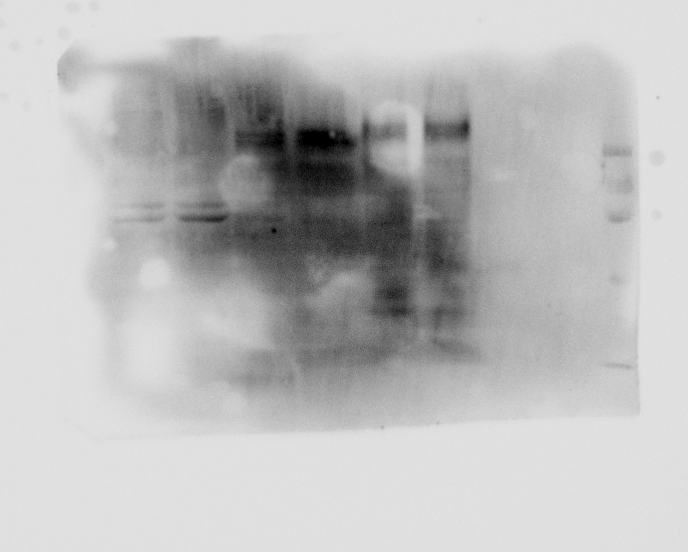

Supplement: Supplemental Information 1 [file peerj-08-9524-s001.zip › WB/AcGFP.tif]

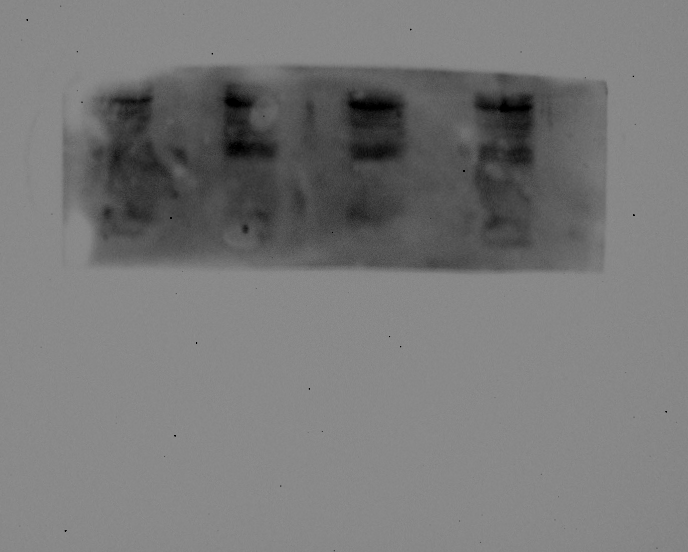

Supplement: Supplemental Information 1 [file peerj-08-9524-s001.zip › WB/CD63.tif]

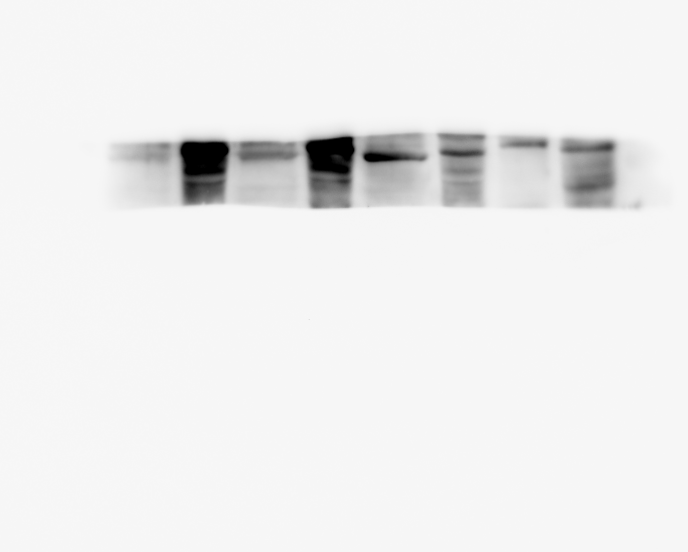

Supplement: Supplemental Information 1 [file peerj-08-9524-s001.zip › WB/flag.tif]

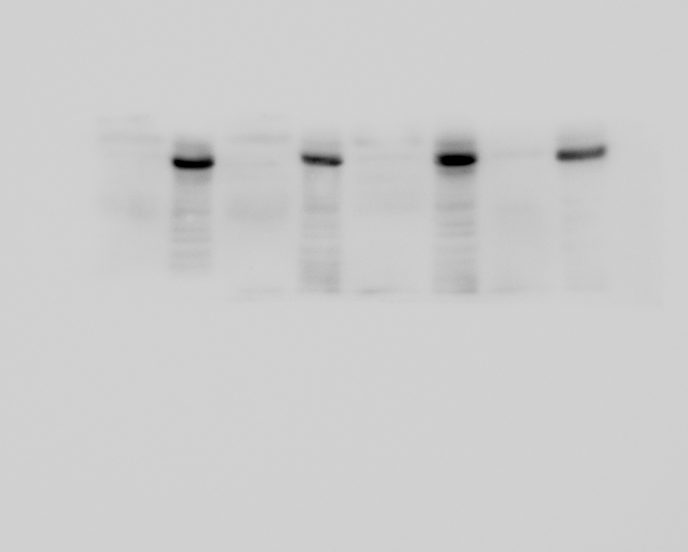

Supplement: Supplemental Information 1 [file peerj-08-9524-s001.zip › WB/gapdh b.tif]

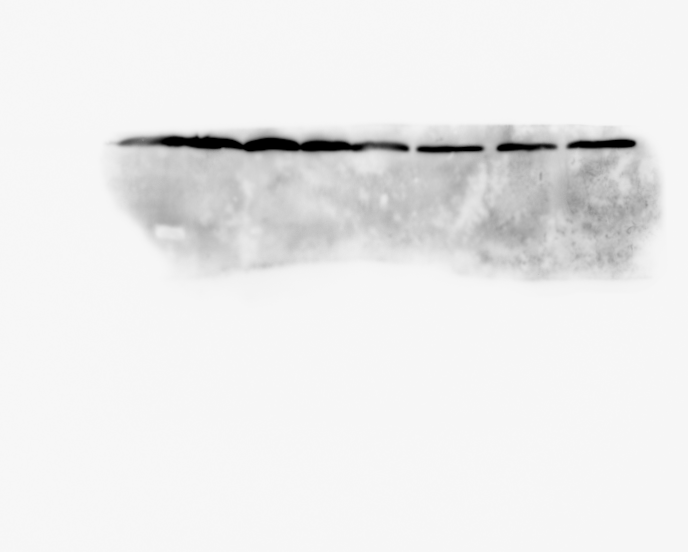

Supplement: Supplemental Information 2 [file peerj-08-9524-s002.zip › GAPDH.tif]

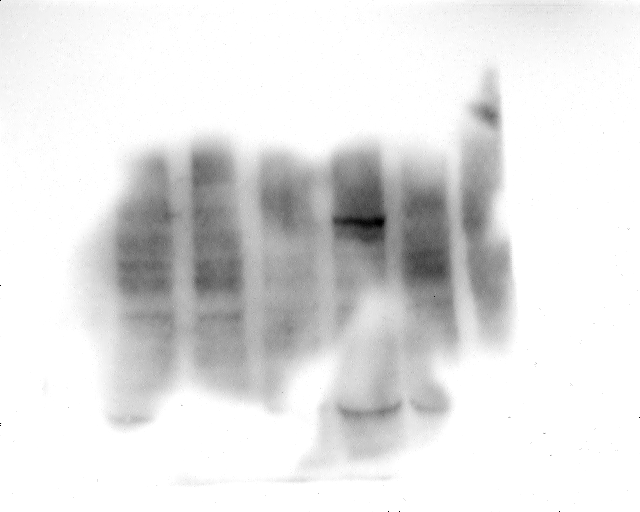

Supplement: Supplemental Information 2 [file peerj-08-9524-s002.zip › GPC3.tif]

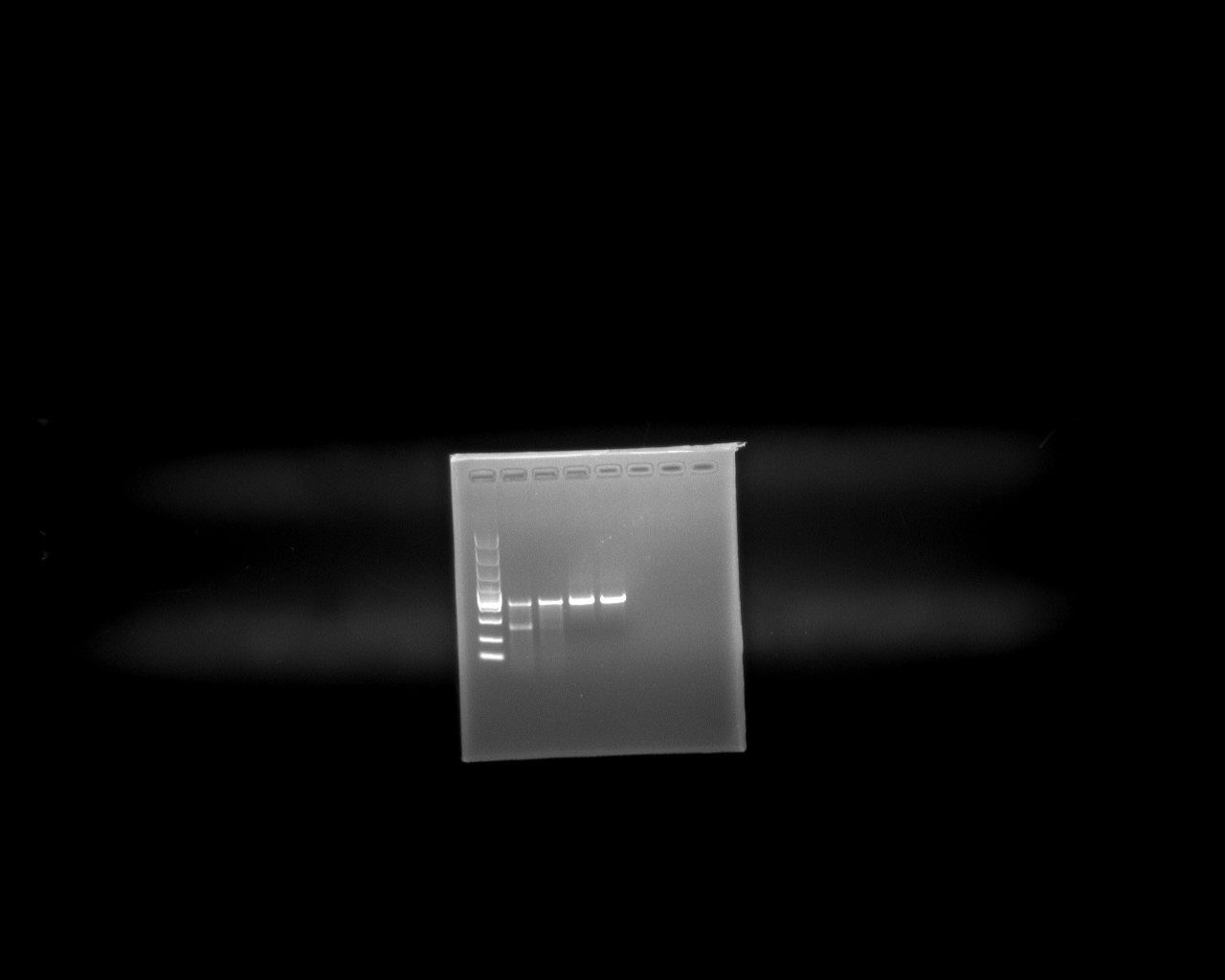

Supplement: Supplemental Information 3 [file peerj-08-9524-s003.zip › Figure 3/A-t7.tif]

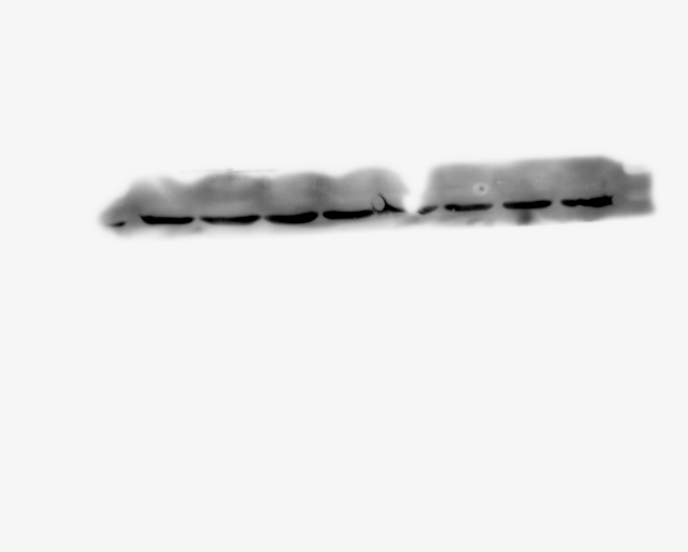

Supplement: Supplemental Information 3 [file peerj-08-9524-s003.zip › Figure 3/B-GAPDH.tif]

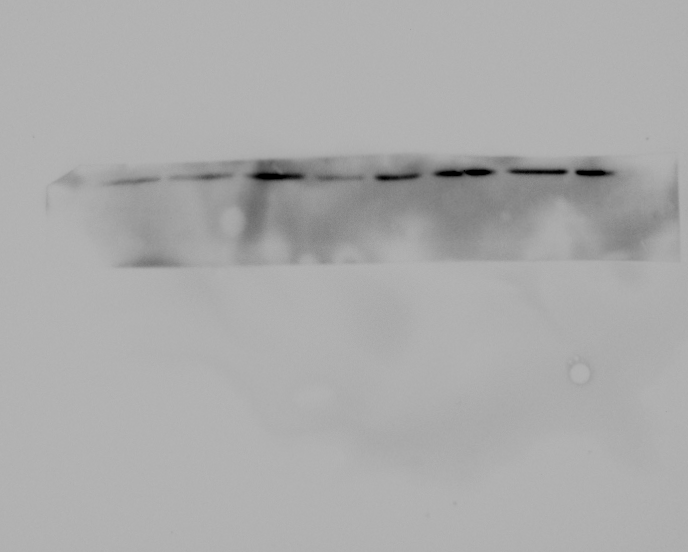

Supplement: Supplemental Information 3 [file peerj-08-9524-s003.zip › Figure 3/B-IQGAP1.tif]

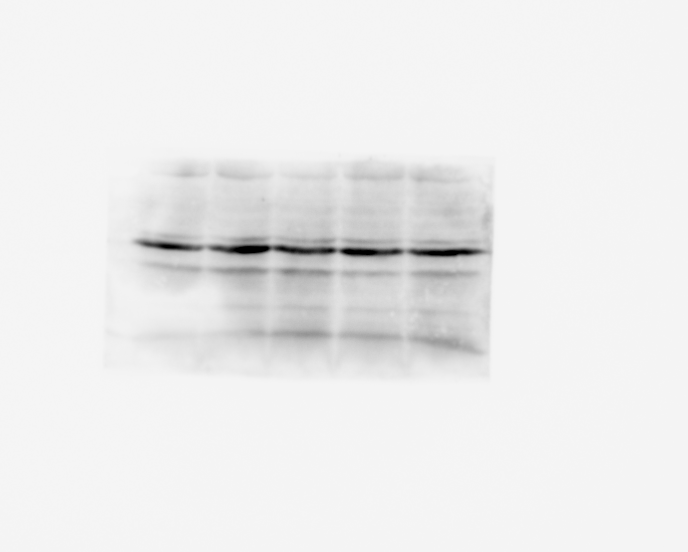

Supplement: Supplemental Information 3 [file peerj-08-9524-s003.zip › Figure 3/C-GAPDH.tif]

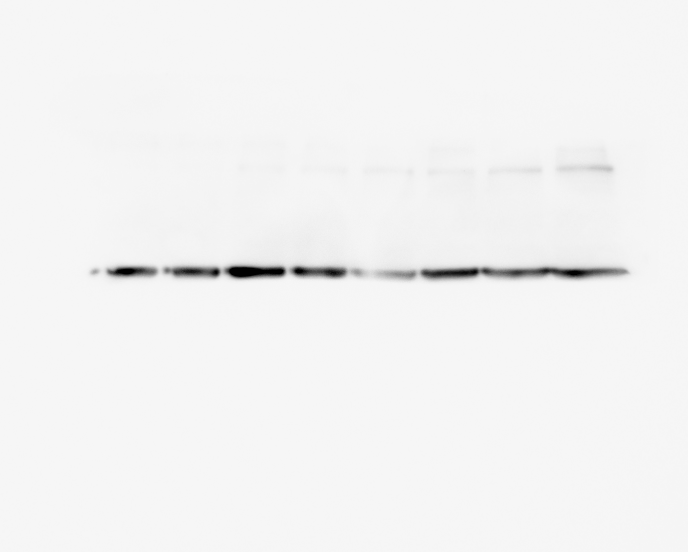

Supplement: Supplemental Information 3 [file peerj-08-9524-s003.zip › Figure 3/C-IQGAP1.tif]

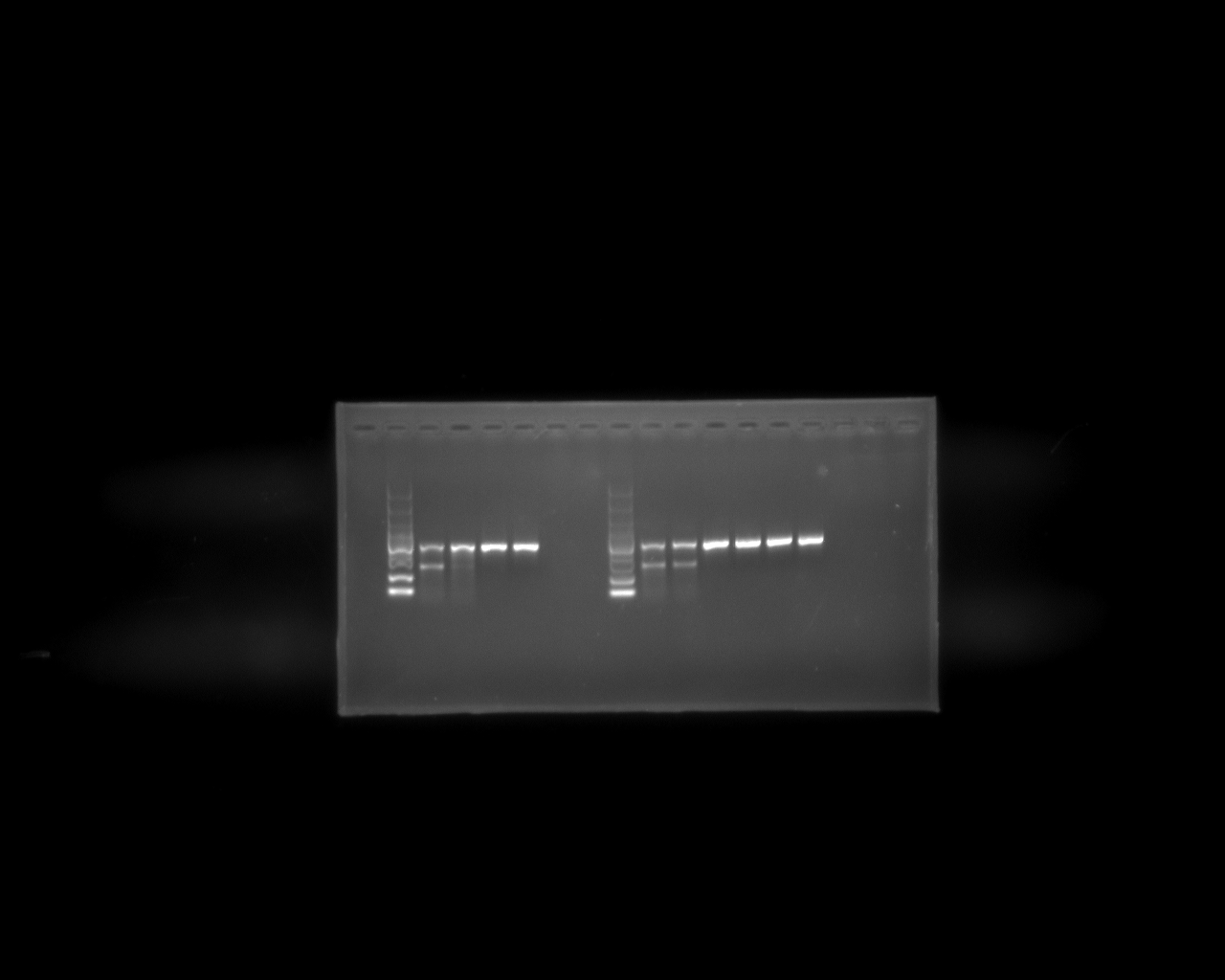

Supplement: Supplemental Information 3 [file peerj-08-9524-s003.zip › Figure 3/D-t7.tif]

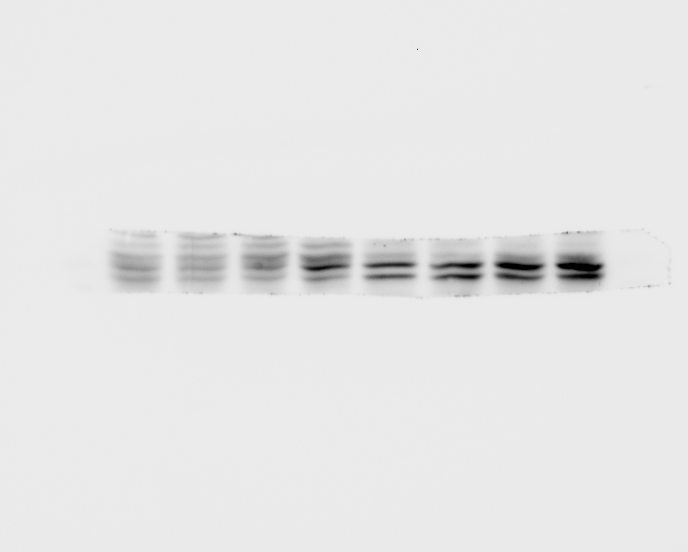

Supplement: Supplemental Information 4 [file peerj-08-9524-s004.zip › BAX.tif]

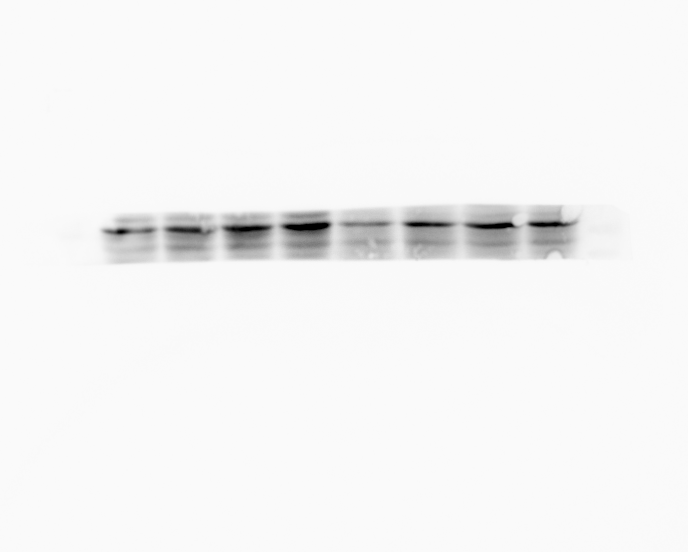

Supplement: Supplemental Information 4 [file peerj-08-9524-s004.zip › BCL2.tif]

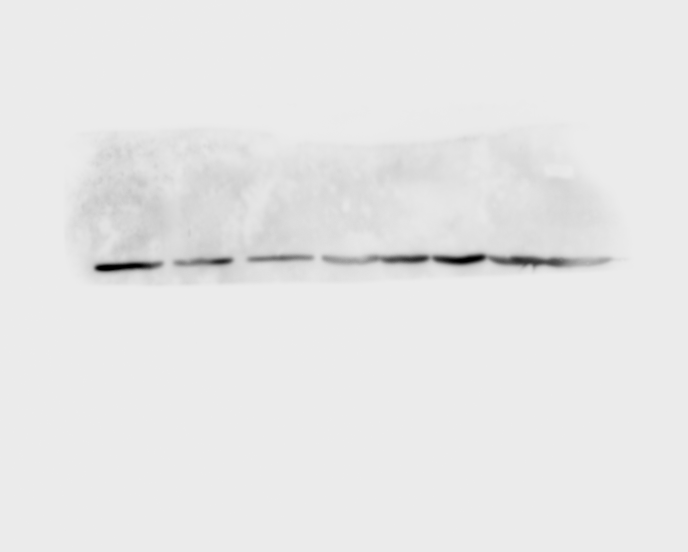

Supplement: Supplemental Information 4 [file peerj-08-9524-s004.zip › CASPASE3.tif]

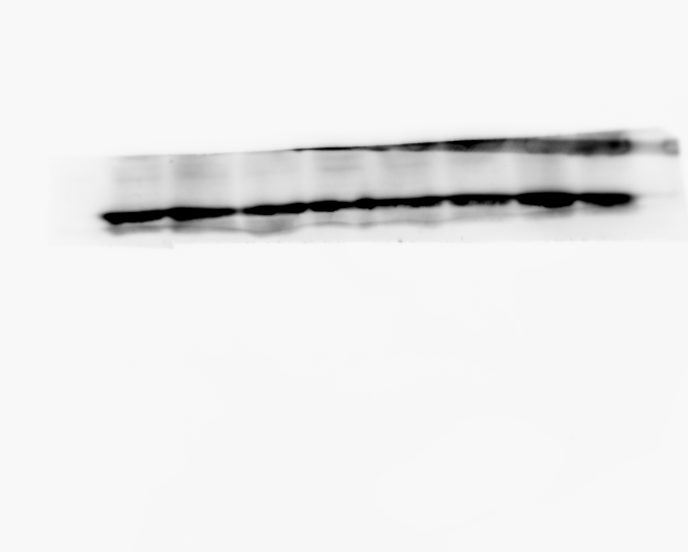

Supplement: Supplemental Information 4 [file peerj-08-9524-s004.zip › GAPDH.tif]

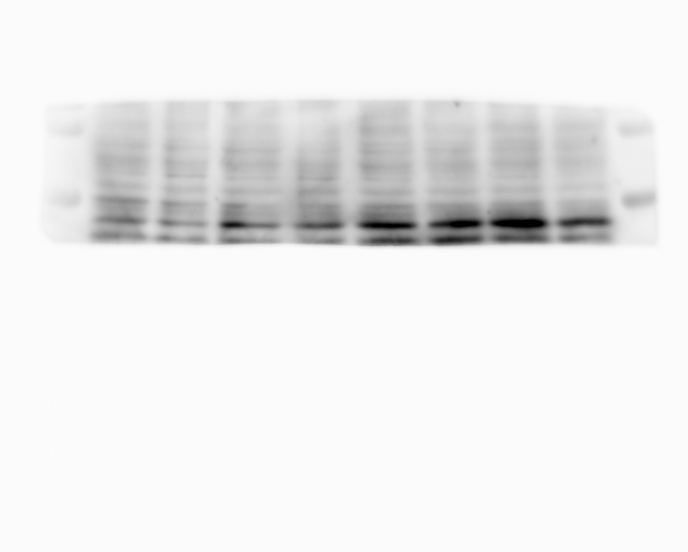

Supplement: Supplemental Information 4 [file peerj-08-9524-s004.zip › IQGAP1.tif]

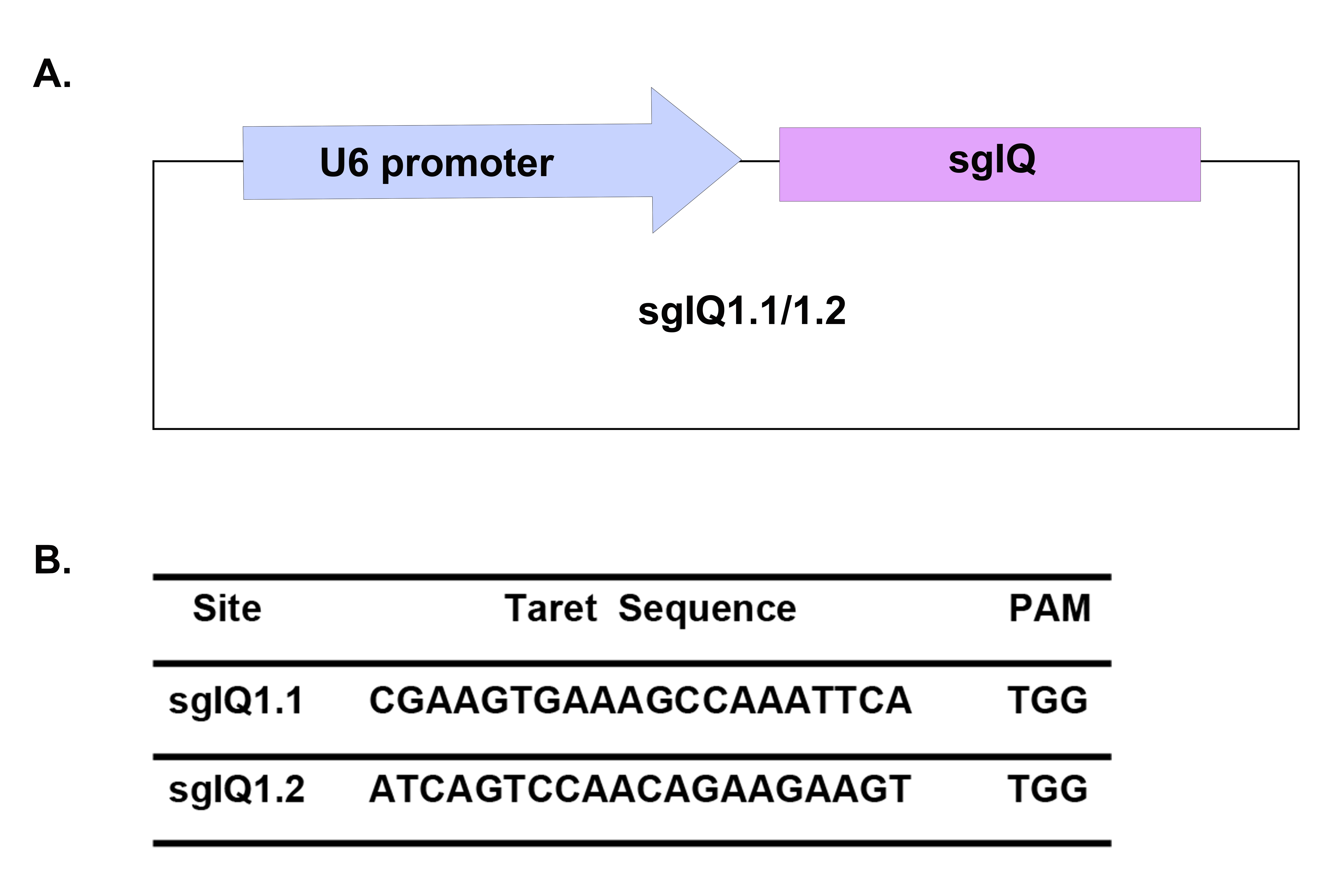

Supplement: Figure S2 — (A) sgIQ 1.1. and sgIQ 1.2 plasmid after cloning of sgIQ 1.1/1.2 sequences into pCas-Guide-GFP plasmid. (C) Sequences of two sgRNAs which were selected to induce Cas9:sgRNA -mediated indels. [file peerj-08-9524-s008.png]
