# Supplementary material for: Engineering of HN3 increases the tumor targeting specificity of exosomes and upgrade the anti-tumor effect of sorafenib on HuH-7 cells
Source: PeerJ. 2020 Jul 20;8:e9524. doi: 10.7717/peerj.9524 (PMC7527773; doi:10.7717/peerj.9524)
Supplement: Supplemental Information 5 [file peerj-08-9524-s005.docx]

**Supplementary Methods**

*Plasmid construction and stable cell line generation*

Sequence encoding LAMP2 signal peptide, HN3 antibody, LAMP2 (segment after the signal peptide) and a ﬂexible peptide linker (GGGGS)_3_ were directly fused into pLVX-AcGFP-N1 in series by homologous recombination to generate HN3-LAMP2-AcGFP plasmid (Figure S1). LAMP2-AcGFP plasmids without HN3 antibody sequence was also prepared to be used as control. Cas9-/sgRNA plasmid was generated by removing Cas9 encoding sequence from pCas-Guide-GFP (Origene, Cat # GE100012), by inserting the EcoRI restriction site before and after the Cas9 encoding gene using the site-directed mutagenesis kit (Vazyme, China). Later, sgIQGAP 1.1 and sgIQGAP 1.2 sequences (Figure S2) were inserted into the plasmid by following the manufacturer’s instructions and henceforth referred as sgIQ 1.1 and sgIQ 1.2. Primers used in this study are given in Table S1.

To stably express Cas9 in HEK-293 and in HuH-7 cells, the cells were transiently transfected with pLenti-CRISPR-V2 (Cat #52961, Addgene), along with pMDLg/pRRE（Cat #12251, Addgene）, pRSV-Rev (Cat #12253, Addgene) and pCMV-VSV-G (Cat #8454, Addgene) virus packaging plasmids. The cells were then screened with 10 µg/mL of puromycin for five passages to get pure lines of Cas9 expressing cells and denoted as C9-293 and C9HuH-7 cells, respectively.

Later, Cas9 expressing HEK-293 cells were again stably transduced with pLVX- HN3-LAMP2-AcGFP and pLVX-LAMP2-AcGFP, screened with 10 µg/mL of blasticidin for five passages to get pure lines of HN3-LAMP2-AcGFP and LAMP2-AcGFP, denoted as HN3LC9-293 and LC9-293 cells respectively. To differentiate GPC3^+^ HuH-7 cells from GPC3^-^ LO2 cells in a co-culture model, HuH-7 cells were stably expressed with mcherry fluorescence protein.
