# Supplementary material for: Engineering of HN3 increases the tumor targeting specificity of exosomes and upgrade the anti-tumor effect of sorafenib on HuH-7 cells
Source: PeerJ. 2020 Jul 20;8:e9524. doi: 10.7717/peerj.9524 (PMC7527773; doi:10.7717/peerj.9524)
Supplement: Table S1 [file peerj-08-9524-s006.docx]

**Table S1.** Primers used in this study

| Primer | Sequence (5’ – 3’) | Use |
| --- | --- | --- |
| C9 Cut M1 | F – GCCGCCGCGGAATTCATGGATAAGAAATACTCAATA  GGACTG  R - CTTATCCATGAATTCCGCGGCGGCAGATCTCCTCGG  TACCGG | To create EcoRI site before Cas9 in pCas-guide-GFP to prepare Cas9-/sgRNA vector |
| C9 Cut M2 | F - CAGCTGGGAGAATTCCCCAAGAAAAAACGCAAGGTG  R - TTTCTTGGGGAATTCTCCCAGCTGACTCAAATCAAT | To create EcoRI site after Cas9 in pCas-guide-GFP to prepare Cas9-/sgRNA vector |
| Eco Del 1 C9 | F - GGGCGGCCGGGCATTCGTCGACTGGAACCGGTACCG  AGG  R - TCGACGAATGCCCGGCCGCCCTATAGTGAGTCGTAT  TAC | To delete EcoRI site in pCas-guide-GFP |
| Eco Del 2 C9 | F - GCAGTTAACGCATTCCCCAGTGGAAAGACGCGCAGG  CAA  R - ACTGGGGAATGCGTTAACTGCCATCCAGCTGATATC  CCC | To delete EcoRI site in pCas-guide-GFP |
| sgIQGAP1.1 | F - GATCGCGAAGTGAAAGCCAAATTCAG  R - AAAACTGAATTTGGCTTTCACTTCGC | To clone sgIQ 1.1 sequences into pCas-guide-GFP and Cas9-/sgRNA to prepare Cas9/sgIQ 1.1 and Cas9-/sgIQ 1.1 (sgIQ 1.1) |
| sgIQGAP1.2 | F - GATCGATCAGTCCAACAGAAGAAGTG  R - AAAACACTTCTTCTGTTGGACTGATC | To clone sgIQ 1.2 sequences into pCas-guide-GFP to prepare Cas9/sgIQ 1.2 |
| RNA Scaffold | 5’- TAATTTGACTGTAAACACAAA  5’- GCACCGACTCGGTGCCACTTT | To test the successful loading and loading efficiency of sgIQ 1.1 after electroporation |
| IQGAP1  Genome | F - GACATTGCCAGGGATATTCGG  R - GGTAACAAATGTCCCATCAG | To amply genomic DNA fragment of IQGAP1 for T7EI Assay |
| LAMP2 RE | F - GGCCCGGGATCCACCATGGTGTGCTTCCGCCTCTTCC  R - TTGCTACCATGACC ATCAAATTGCTCATATCCAGCATG | To fuse LAMP2 into pLVX-AcGFP plasmid |
| HN3LAMP2 RE | F- GTGCGGTCTTATGCAATGCAGGTGCAGCTGGTGCAGTCTG  R – TTCCAAGCTGCCTCCGCCGCCACTTGAGGAGACGGTGACCAGGG  TTC | To fuse HN3LAMP2 into pLVX-LAMP2-AcGFP plasmid |
